# Engagement With Sports After Solid Organ Transplant

**DOI:** 10.7759/cureus.68622

**Published:** 2024-09-04

**Authors:** Nikita Saji, Arya S Kumar, Jo Joseph, Pavan Kumar, Anandajith Kartha, Kamal Bijlani, Zubair U Mohamed

**Affiliations:** 1 Department of Anaesthesia and Critical Care, Amrita Institute of Medical Sciences and Research Centre, Ernakulam, IND; 2 Department of Infection Control and Epidemiology/Medical Administration, Amrita Institute of Medical Sciences and Research Centre, Ernakulam, IND; 3 Department of Cardiology, Lisie Heart Institute, Lisie Hospital, Kochi, IND; 4 School of Artificial Intelligence, Amrita Vishwa Vidyapeetham, Faridabad, IND

**Keywords:** pancreas and kidney transplant, heart transplantation, pancreas transplantation, living donor kidney transplantation, living donor liver transplant-ldlt, organ donation, rehabilitation, physical activities, sports, solid organ transplantation

## Abstract

Introduction

Transplant Games lend a unique opportunity not only by providing a platform for donors and recipients to engage in sports but also to counter the negativity surrounding organ donation and showcase to the world that transplant recipients can lead active lives. When the Transplant Games were held in Kochi, Kerala, India, for the first time, it provided a venue to engage with transplant recipients, donors, and the families of deceased donors. We aimed to understand the impact that engagement in sports brings on the lives of donors and recipients.

Methods

After obtaining permission from the organizers, we explained the objectives of the survey to the participants and encouraged them to participate. A survey, covering basic demographic information, transplantation details, and questions related to sports engagement was formulated. Participants could complete the survey electronically via a quick response code or in hard copy. They were fully informed about the objectives of the survey and had the right to withdraw at any stage without consequences. The survey was available for five hours during the games. The study received institutional ethics committee approval (ECASM-AIMS-2024-059).

Results

Among the approximate 150 participants, we received 78 respondents (52%). After the nine who withdrew consent were removed, we had a full response from 69 participants. Of these, 59 were males (85.5%), and 10 (14.5%) were females. The average age of the participants was 45 ± 13 years. Self-motivation was the most common factor in taking up sports for 30.4% of the responders, followed by family and friends in 23.2% and transplant doctors in 5.8%. Liver Foundation of Kerala (LIFOK), a self-help group of transplant recipients, played a major role in 4.3% of the responders. Bowling was the most popular sport with 23 mentions, followed by carroms and badminton with 27 and 20 mentions, respectively. Donors started to take an active role in sports earlier than recipients, 3.1 ± 1.89 vs. 5.7 ± 5.5 months. The most common reason cited for taking up sports was to become part of the transplant community, followed by a desire to embrace a healthier lifestyle and improve fitness levels. Although none had a personalized coach, most intensified their training and improved nutrition as part of their preparation for the games.

Conclusion

Our survey is limited by its small and self-selected sample size. Our study highlights the significant role of self-motivation, family support, and self-help groups in encouraging solid organ transplant recipients and donors to engage in sports after surgery. It also highlights the need for more proactive encouragement from doctors and better availability of sports facilities and support staff to help transplant recipients and donors engage in physical activities, which are crucial for their physical and emotional well-being.

## Introduction

In the latter half of the 20th century, Dr. Maurice Slapak, a transplant surgeon, came up with the idea of Transplant Games to counter the negativity surrounding organ donation and showcase to the world that transplant recipients can lead active lives. The first Transplant Games were held in Portsmouth, UK [1]. Transplant Games have, subsequently, become a global phenomenon, providing a platform for donors and recipients to engage in sports, fostering a sense of community and mutual support [2].

There is a growing understanding of the importance of lifestyle factors in post-transplant care, as regular physical activity has been associated with improved outcomes, including decreased lifestyle-associated diseases [3,4]. Understanding the experiences of donors and recipients while engaging in sports after transplantation could provide valuable insights into the challenges they face and how this could be addressed.

Although India currently ranks third globally in the number of transplants performed, the unique challenges faced by this patient population in taking up sports after a life-changing surgery have not been studied in India before [5]. On December 9, 2023, Heart Care Foundation, India, in collaboration with government and non-government organizations, organized the Transplant Games in Kochi, Kerala, India, which provided a unique opportunity to engage with transplant recipients, donors, and the families of deceased donors [6]. We aimed to understand the impact that engagement in sports brings on the lives of donors and recipients and to understand the shared experiences and unique challenges faced by both groups.

This study was partly presented as a meeting abstract at the 2024 Annual Conference of the Indian Society of Critical Care Medicine on March 2, 2024.

## Materials and methods

The Transplant Games was held on a single day (December 9, 2023) over three venues - Regional Sports Centre (RSC), Kochi; Jawaharlal Nehru Stadium, Kochi; and Lulu Mall, Kochi. The games included a total of 10 events, of which 5,000 m run and 200 m dash were held at Jawaharlal Nehru Stadium, bowling at Lulu Mall, and the rest of the events (badminton, basketball shoot out, chess, golf, swimming, dart, carroms, and table tennis) at RSC. We aimed to understand the impact that engagement in sports brings on the lives of donors and recipients.

A survey covering basic demographic information, transplantation details, and questions related to sports engagement was formulated. The survey was made available in both English and Malayalam, the local language of Kerala, India. After obtaining permission from the organizing committee, two authors (ZUM and ASK) visited the venue, explained the objectives of the survey to the participants, and encouraged them to participate, emphasizing the importance of their contribution to advancing knowledge in the fields of organ transplantation and sports. They were fully informed about their right to withdraw at any stage without consequences. They were given the option to either complete the survey electronically via a quick response (QR) code or in hard copy, but not both. The QR code was displayed at multiple sites in the venue, and the survey was available for five hours during the games. Participants' email addresses and phone numbers were collected to avoid duplication of data. Due to logistical constraints, only the participants at RSC were invited to participate. The study received institutional ethics committee approval (ECASM-AIMS-2024-059). The data of the electronic and hard copies were collected, and descriptive analysis was performed on Microsoft Excel (Microsoft® Corporation, Redmond, WA).

## Results

Among the approximately 150 participants who partook in the event at RSC, we received 78 respondents (52%). After the nine who withdrew consent were removed from the analysis, we had full responses from 69 participants. The details of the participants are presented in Table 1.

**Table 1:** Demographic details of the participants

Category	Data
Gender	
Male	59 (85.5%)
Female	10 (14.5%)
Population distribution	
Keralites	67 (97.1%)
Non-Keralites	2 (2.9%)
Average age at the time of transplant	40.7 ± 12.6 years
Types of transplant	
Liver	22 (41.5%)
Kidney	26 (49.1%)
Heart	3 (5.7%)
Kidney-pancreas	2 (3.8%)
Patients with >1 transplant	6 (11.3%)
Participation in sports before their transplant/donation	
Participated	42 (60.9%)
Not participated	27 (39.1%)
Main motivation to take up sports	
Self-motivation	21 (30.4%)
Family and friends	16 (23.2%)
Liver Foundation of Kerala (LIFOK)	3 (4.3%)
Doctors	4 (5.8%)
Popular sports	
Bowling	23 (33.33%)
Carroms	26 (37.68%)
Badminton	20 (29%)
Start of active role in sports post-surgery	
Donors	3.1 ± 1.89 months
Recipients	5.7 ± 5.5 months
Advice regarding exercise post-surgery	
Donors	73%
Recipients	62%

Among the 69 participants who completed the questionnaire, 59 were males (85.5%), and 10 (14.5%) were females. Six responders received more than one transplant. Transplant recipients were 53/69, of which six were females. This included three heart, 26 kidney, 22 liver, and two kidney-pancreas recipients. All the participants were Indians, and only two were from outside Kerala. The average age of the participants was 45 ± 13 years. The transplant recipients had an average age of 40.7 ± 12.6 years at the time of transplant. One-third of the participants did not partake in sports before their surgery.

Self-motivation was the most common factor in taking up sports for 30.4% of the responders, followed by family and friends in 23.2% and transplant doctors in 5.8%. LIFOK, a self-help group of transplant recipients, played a major role in 4.3% of the respondents. Bowling was the most popular sport with 23 mentions, followed by carroms and badminton with 27 and 20 mentions, respectively.

Donors started to take an active role in sports earlier than recipients, 3.1 ± 1.89 vs. 5.7 ± 5.5 months. The most common reason cited to take up sports was to become part of the transplant community, followed by a desire to embrace a healthier lifestyle and improve fitness levels. Six out of 69 (8.69%) donors responded that they took up sports to compete nationally and break records; 62% of recipients and 73% of donors received advice regarding exercise after surgery - mostly from nutritionists and physiotherapists. While none had a personalized coach, most intensified their training and improved nutrition as part of their preparation for the games. Most recipients and donors were able to train one to three days a week for at least 30 minutes, with 20% of recipients training for six to seven days a week. Among the recipients, 61.9% of males and 100% of females responded that they felt confident in their ability to train with the same intensity as a non-transplanted healthy counterpart.

## Discussion

During the course of the last five decades, Transplant Games have served not just as a sporting event but also as a platform for enhancing well-being and providing support for producing health-promoting environments among transplant recipients and donors [7]. This is the first report from India that studied the engagement with sports among solid organ transplant recipients and donors.

The importance of Transplant Games in improving not only physical fitness but also psychological well-being and quality of life has been studied earlier [8,9]. Family and friends, as well as self-help groups like LIFOK, played a significant role in encouraging participants to take up sports, highlighting the importance of social support in the post-transplantation recovery process. Self-motivation emerged as an important factor and despite not having personalized coaches, most participants intensified their training, demonstrating their commitment to sports and the event. Similar findings have been reported by D'Ambrosio et al., who showed that pre-games training programs not only helped to motivate participation and achieve higher training intensities but also fostered social interaction [10]. Most transplant recipients expressed confidence in their ability to train and be on par with non-transplanted individuals.

One significant insight from our study is that doctors do not seem to encourage patients to participate in sports. This may be a reflection of the doctor-patient relationship being more focused on organ-specific recovery while the other healthcare workers focus on holistic recovery [4]. It is possible that doctors advising and encouraging a healthier lifestyle, including sports, could have a higher acceptance among patients while other healthcare workers build upon this to continue their education, rehabilitation, and training.

Our games provided a unique opportunity to engage with not only transplant recipients but also living donors and the families of deceased donors. Despite India being at the forefront in terms of transplant numbers, especially from living donors, little has been published regarding the rehabilitation of living donors. Worldwide literature highlights many facets of donor care that need to improve, including long-term health and allaying economic, social, and emotional burdens [11]. A high incidence of depression and anxiety among living donors, especially among females, has been observed within the first three months after donation [12]. Interestingly, the mean time taken by donors before engaging in sports was 3.1 months. Whether earlier engagement, addressing the social, economic, and emotional burden among donors, could rehabilitate them faster is hypothesis generating. Our questionnaire was not designed to explore this topic further.

Despite the worldwide efforts to improve deceased donation, waiting lists continue to get longer, and the role of living donation in kidney, liver, and even lung transplantation continues to be redefined [13-15]. On both sides of the Atlantic, there is a re-kindling of the discussion on re-aligning the donor-recipient relationship from being purely altruistic to a reciprocal and mutually beneficial one [16,17]. Irrespective of how this narrative evolves, the transplant community should address the social, emotional, economic, and holistic well-being of the donor better. A centralized registry of living liver donors, similar to the one proposed by the Liver Transplant Society of India, is perhaps a good platform to define and study the barriers and promote the enablers.

## Conclusions

While our survey is limited by its small and self-selected sample size, with a majority of participants from Kerala, our findings, in general, echo the previously reported sentiments and enthusiasm of the transplant patient community from other parts of the world. It also highlights certain important areas that need further attention in India. Firstly, it draws our attention to the minimal encouragement and motivation given regarding engagement with sports to the transplant community by the doctors in the field of transplantation. Secondly, we are informed regarding the dearth of sporting facilities available to this patient population. Thirdly, it mirrors the urgent worldwide need to address the long-term holistic care plan that living donors deserve.
